# Evaluation of zona pellucida birefringence intensity during in vitro maturation of oocytes from stimulated cycles

**DOI:** 10.1186/1477-7827-9-53

**Published:** 2011-04-23

**Authors:** Claudia G Petersen, Laura D Vagnini, Ana L Mauri, Fabiana C Massaro, Liliane FI Silva, Mario Cavagna, Ricardo LR Baruffi, Joao BA Oliveira, José G Franco

**Affiliations:** 1 Department of Gynecology and Obstetrics, Botucatu Medical School, São Paulo State University - UNESP, Botucatu, Brazil; 2 Center for Human Reproduction Prof. Franco Jr, Ribeirao Preto, Brazil; 3 Paulista Center for Diagnosis, Research and Training, Ribeirao Preto, Brazil

## Abstract

**Background:**

This study evaluated whether there is a relationship between the zona pellucida birefringence (ZP-BF) intensity and the nuclear (NM) and cytoplasmic (CM) in vitro maturation of human oocytes from stimulated cycles.

**Results:**

The ZP-BF was evaluated under an inverted microscope with a polarizing optical system and was scored as high/positive (when the ZP image presented a uniform and intense birefringence) or low/negative (when the image presented moderate and heterogeneous birefringence). CM was analyzed by evaluating the distribution of cortical granules (CGs) throughout the ooplasm by immunofluorescence staining. CM was classified as: complete, when CG was localized in the periphery; incomplete, when oocytes presented a cluster of CGs in the center; or in transition, when oocytes had both in clusters throughout cytoplasm and distributed in a layer in the cytoplasm periphery Nuclear maturation: From a total of 83 germinal vesicle (GV) stage oocytes, 58 of oocytes (69.9%) reached NM at the metaphase II stage. From these 58 oocytes matured in vitro, the high/positively scoring ZP-BF was presented in 82.7% of oocytes at the GV stage, in 75.8% of oocytes when at the metaphase I, and in 82.7% when oocytes reached MII. No relationship was observed between NM and ZP-BF positive/negative scores (*P *= 0.55). These variables had a low Pearson's correlation coefficient (r = 0.081). Cytoplasmic maturation: A total of 85 in vitro-matured MII oocytes were fixed for CM evaluation. Forty-nine oocytes of them (57.6%) showed the complete CM, 30 (61.2%) presented a high/positively scoring ZP-BF and 19 (38.8%) had a low/negatively scoring ZP-BF. From 36 oocytes (42.3%) with incomplete CM, 18 (50%) presented a high/positively scoring ZPBF and 18 (50%) had a low/negatively scoring ZP-BF. No relationship was observed between CM and ZP-BF positive/negative scores (*P *= 0.42). These variables had a low Pearson's correlation coefficient (r = 0.11).

**Conclusions:**

The current study demonstrated an absence of relationship between ZP-BF high/positive or low/negative score and nuclear and cytoplasmic in vitro maturation of oocytes from stimulation cycles.

## Background

When the oocyte is preparing for ovulation and interaction with spermatozoon, a series of changes occur inside of this cell. This is a complex process that includes nuclear, meiotic and cytoplasmic events [1]. To reach optimal competence for further development, cytoplasmic oocyte maturation (CM) must occur in synchronization to nuclear maturation (NM). The zona pellucida (ZP) is a unique extracellular coat that surrounds the maturing oocyte during ovulation, fertilization and early embryonic development [2]. The ZP is a multilaminar glycoprotein coat composed of filaments, which are forming a three-dimensional network structure that shows a high birefringence [3,4]. The observation of ZP birefringence (ZP-BF) intensity with a polarized microscope has been proposed to identify oocyte competence and subsequent ART outcomes. A high/positively scoring ZP-BF has been significantly associated with better embryo quality and implantation and pregnancy rates when compared with oocytes showing a low/negatively scoring ZP-BF [5-7]. According to Montag et al. [5], a high/positively scoring ZP-BF probably reflects a healthy oocyte with full nuclear and cytoplasmic maturation.

Our objective in this study was to evaluate whether there is a relationship between the NM and CM of in vitro maturated human oocytes from stimulated cycles and the ZP-BF intensity of their zona pellucidae.

## Methods

### Participants

This prospective study was conducted on 92 patients who were admitted to an intracytoplasmic sperm injection (ICSI) program at the Center for Human Reproduction Prof. Franco Jr, Ribeirão Preto, Brazil. According to a protocol approved by the Center's Institutional Ethics Committee, the population included in this study was comprised of patients, which had at least 6 oocytes in MII before the ICSI procedure, and patients which had all immature oocytes (germinal vesicles; GV) used for the in vitro evaluation of NM and CM.

### Study design

In the first experiment, a total of 83 GV oocytes obtained from 38 patients were cultured in P1 medium (Irvine Scientific, Santa Ana, CA, USA) supplemented with 10% human serum albumin (HSA) for 48 hours to allow them to complete NM. During NM, the ZP-BF intensities of oocytes were evaluated at the MI and MII stages. A total of 58 GV oocytes completed NM and reached the MII stage.

In the second experiment, a total of 164 GV oocytes (the 83 GV oocytes included in group I plus 81 new GV oocytes) obtained from 54 patients (38 from group I plus 16 new patients) were cultured in P1 medium supplemented with 10% HSA. Eghty five of oocytes presented the first polar body after 48 hours of culture. These 85 MII oocytes were evaluated for ZP-BF intensity and fixed for the evaluation of CM. As 68.2% (58/85) of MII oocytes were originated from the group I population, the major part of the oocytes presented syncronization in term of NM and CM.

### Ovarian stimulation

The study patients underwent ovarian stimulation. First, pituitary down-regulation was started during the second phase of the previous menstrual cycle with the GnRH-a nafarelin acetate at a dose of 400 μg/day for 14 days (Synarel; Pfizer, SP, Brazil). Then, ovarian stimulation was started with rFSH (Gonal F; Serono, SP, Brazil) at fixed dose of 150 to 225IU with rLH (Luveris; Serono, SP, Brazil) at 75 IU/day for a period of 7 days. On the 8th day of ovarian stimulation, the rFSH dose was adapted according to ovarian response, and rLH supplementation was increased to 150 IU/day when one or more follicles measuring ≥10 mm in diameter were found. When one or more follicles measuring ≥17 mm in diameter were observed, 250 μg rhCG (Ovidrel; Serono, SP, Brazil) was administered. Oocyte retrieval was performed after 36 hours of rhCG administration by transvaginal ultrasound-guided aspiration.

### Preparation of oocytes

All the retrieved oocytes were incubated in culture medium (P1; Irvine Scientific, Santa Ana, CA, USA) at 37°C and 5.5% CO_2 _for 1 hour. Cumulus cells were removed by exposing the acolytes for 30 sec to modified human tubal fluid medium (mHTF, Irvine Scientific, Santa Ana, CA, USA) containing 80 IU/ml hyaluronidase (Irvine Scientific, Santa Ana, CA, USA), after which coronal cells were manually removed using a stripper (Cook, Australia). The denuded oocytes were classified according to their level of maturation. Oocytes with the first polar body, i.e., at the MII stage, were considered as mature and used for the ICSI procedure. All those in the GV stage were considered as immature and used for this study. The oocytes maturation was checked between 24 to 48hours. As not all reached maturation by 24 hours, the ZP evaluation of all the mature oocytes was performed in a defined time, i.e past 48 hours of culture. After ZP evaluation, the oocytes were fixated for further CM evaluation.

### Zona pellucida imaging

For the ZP-BF evaluation, the oocytes were placed individually in a 5 μl drop of buffered medium (modified HTF; Irvine Scientific, Santa Ana, CA, USA) in a glass culture dish (FluoroDish, World Precision) covered with mineral oil that was previously equilibrated at 37°C. The dish containing the oocytes was placed under an inverted microscope (Eclipse TE300; Nikon, Tokyo, Japan) equipped with a stage heated to 37°C (Tokai Hit, Tokyo, Japan) and a polarizing optical system (OCTAX PolarAIDE; Octax, Herborn, Germany). The OCTAX PolarAIDE system consists of 6 components: a green filter, a circular polarizer, a polarizing 20X objective, a liquid crystal slider, a USB 2.0 digital camera and an imaging software module (OCTAX EyeWare). This system displays BF structures on the computer monitor using the OCTAX EyeWare software.

**Figure 1:**
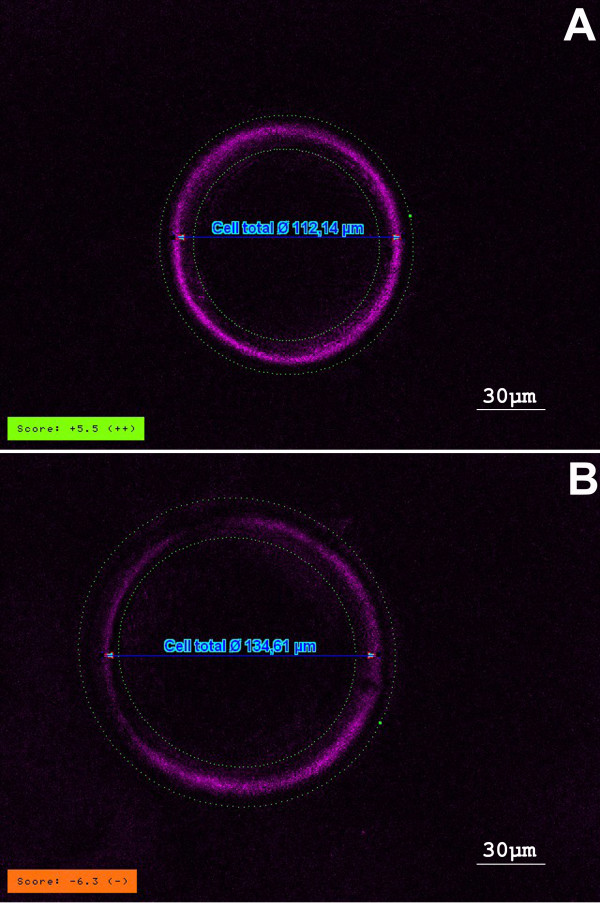
**Zona pellucida birefringence intensity**. A. high/positive score; B.low/negative score. OCTAX PolarAIDE system

Polarization microscopy analysis of the ZP of human oocyte reveals a three-layered sctructure where only the inner layer has birefringent properties. Once detected, the OCTAX PolarAIDE system quantifies (giving a number) the intensity of the birefringence of the inner layer, taking values from many points around the inner zona layer. An algorithm deduces a score from these values [5,8]. Therefore, in this study, the ZP-BF was classified as high when the score was positive (i.e., score number >0; the ZP image presented a uniform and strong coloration around its surface; Figure 1A). The ZP-BF was low when the score was negative (i.e., score number <0; the ZP image presented a light color without uniformity on its surface; Figure 1B).

### Oocyte fixation and cortical granules staining

Coloration of the cortical granules (CGs) was performed using a modified method described by Cherr et al. [9]. After NM confirmation, the ZPs were removed with 0.5% pronase diluted in phosphate buffered saline (PBS) for 5 min at 37°C. The oocytes without the ZP were then fixed for 30 min at room temperature in 3% formaldehyde diluted in PBS and kept at 4°C in a blocking solution (BS) for 1 to 5 days. The BS consisted of 1 mg/ml of bovine serum albumin (BSA), 100 mM glycine and 0.2% sodium azide diluted in PBS. For the permeabilization, the oocytes were incubated in a BS containing 0.1% Triton X-100 at 38°C for 2 minutes. The oocytes were then incubated in 10 μg/ml of Lens culinaris agglutinin conjugated to fluorescein isothiocyanate (FITC) in BS for 15 min (the Lens culinaris agglutinin lectin links specifically to the alpha D-mannose present in the CGs). The oocytes were washed 3 times in BS and stained with 10 μg/ml of Hoechst 33342 in BS for 10 min. The oocytes were transferred to 1 mg/ml of polyvinylpyrrolidone (PVP) solution in PBS for 1 min, deposited into 2 μl glycerol on the slides and fixed between the slide and coverslip. CM evaluation was performed using the fluorescence microscopy (Olympus BX50, Tokyo, Japan). A single observer, blinded to subject identity and the results of ZP evaluation, interpreted the fluorescence colour shades to rule out inter-technician variability. To determine intra-technician variability for immunofluresence reading, slides were made from randomly selected donors to analyse the cytoplasmic maturation. The slides without any identification were read at least three times and the intra-individual variability for the percentages of oocyte with complete or incomplete citoplasmic maturation was ≈2%. In addition, negative control was included (i.e. omitted the incubation with Lens culinaris). It should be noted that due to the difficult of having the oocytes for this trial (immature oocyte from stimulated cycle), the intra-technician variability and negative control were carried only with oocytes from donors before the analysis of the study.

### Cytoplasmic maturation classifications

**Figure 2:**
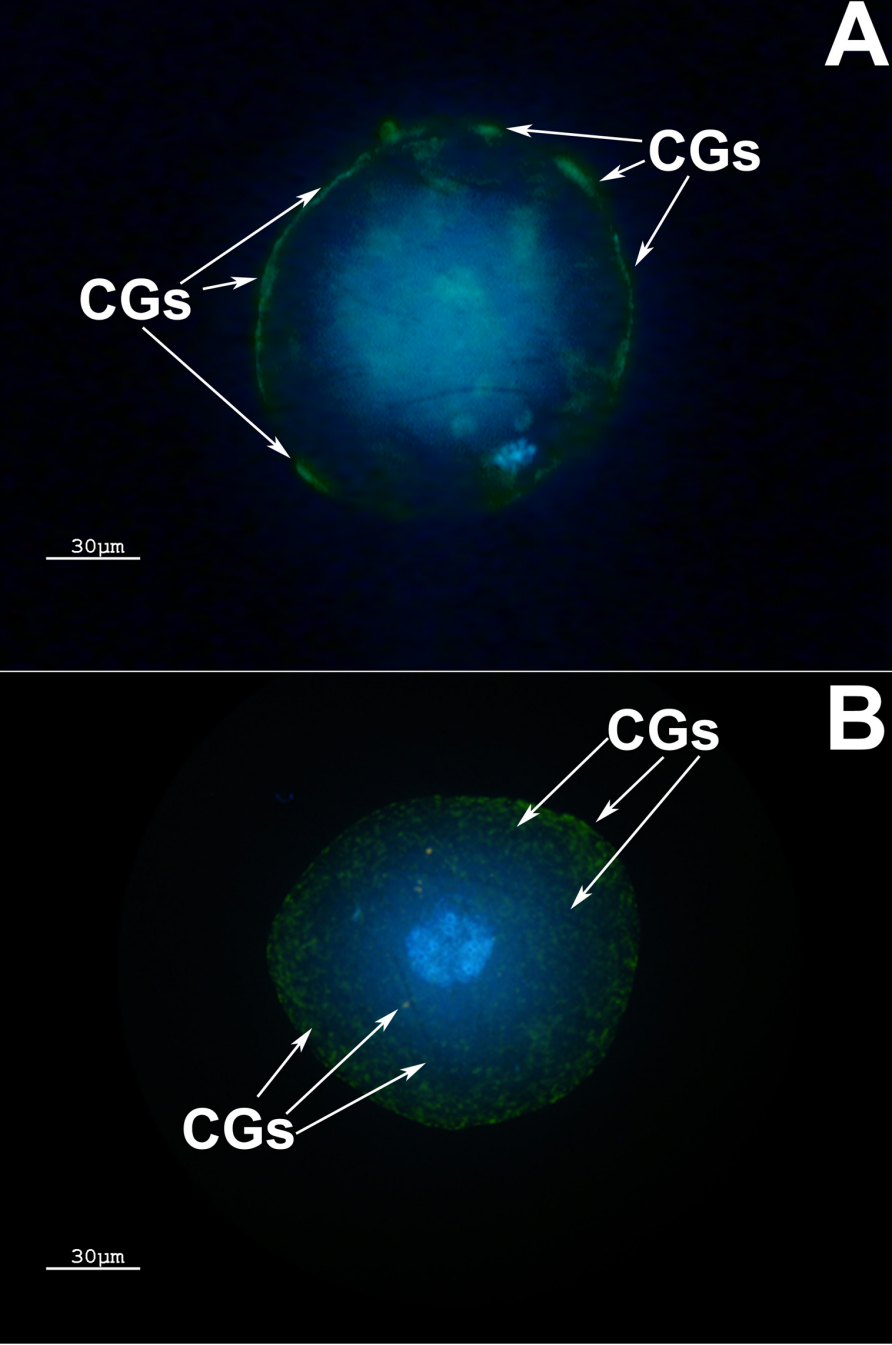
**Cytoplasmic Maturation**. (A) Complete cytoplasmic maturation: CGs are located on the MII oocyte periphery. (B) Incomplete cytoplasmic maturation: CGs are both clustered in the center and on the periphery of the MII oocyte. CGs = cortical granules

CM was classified as complete (oocytes with all the CGs on the oocyte periphery; Figure 2A) or as incomplete (oocytes presenting CGs in cluster throughout cytoplasm or presenting both clusters and a layer in the oocyte periphery; Figure 2B).

### Statistical Analysis

Data were analyzed using InStat version 3.0 (GraphPad Software, San Diego, CA, USA) on a MacIntosh computer (Apple Computer, Inc., Cupertino, CA, USA). The numerical values of the ZP scores were reported as mean ± standart deviation (μ ± SD). The chi-squared test, Mann-Whitney test, Kruskal-Wallis test and Pearson's correlation coefficients were calculated when appropriate. The significance level was set at *P *< 0.05.

## Results

### Nuclear maturation

From a total of 83 GV oocytes, 58 oocytes (69.9%) reached NM (MII stage). From these 58 oocytes matured in vitro, a high/positively scoring ZP-BF was presented in 82.7% of GV oocytes, 75.8% of MI oocytes, and 82.7% of MII oocytes. The association between NM and ZP-BF high/positive and low/negative scores was not statistically significant (*P *= 0.55). In addition, these variables showed a low Pearson's correlation coefficient (r = 0.081). Table 1 summarizes the results.

**Table 1:** The correlation between nuclear maturation (NM) in GV, MI, and MII oocytes and zona pellucida birefringence intensity (ZP-BF)

Oocyte stage Score	ZP-BF Score		Total
	high/positive	low/negative	
GV	48 (82.8%)	10 (17.2%)	58
MI	44 (75.9%)	14 (24.1%)	58
MII	48 (82.8%)	10 (17.2%)	58

Chi-squared value = 1.17 *P *= 0.55; Pearson's correlation coefficient r = 0.081

The analysis using the numerical values of the scores did not observe significant difference in ZP-BF among GV (2.4 ± 4.5), MI (3.0 ± 4.6) and MII (3.3 ± 4.3) oocytes (*P *> 0.05). Similarly, the analysis among subgroups the numerical values of the scores also did not find significant diference: subgroup of oocytes that present high/positive ZP-BF(*P *> 0.05): GV = 4.0 ± 3.3, MI = 4.7 ± 3.3 and MII = 4.8 ± 3.5; subgroup of oocytes that present low/negative ZP-BF(*P *> 0.05): GV = -3.8 ± 2.6, MI = -3.4 ± 2.8 and MII = -3.0 ± 1.5. Table 2 summarizes the results.

**Table 2:** The correlation between nuclear maturation (NM) in GV, MI, and MII oocytes and zona pellucida birefringence intensity (ZP-BF)

Oocyte stage Score	ZP-BF Score		
	total	high/positive	low/negative
GV	2.4 ± 4.5*	4.0 ± 3.3^§^	-3.8 ± 2.6^¶^
MI	3.0 ± 4.6*	4.7 ± 3.3^§^	-3.4 ± 2.8^¶^
MII	3.3 ± 4.3*	4.8 ± 3.5^§^	-3.0 ± 1.5^¶^

The analysis using the numerical values of the ZP scores. Data are reported as means ± SD.

*P > 0.05; ^§^*P *> 0.05; ^¶^*P *> 0.05

### Cytoplasmic maturation

From a total of 85 MII oocytes matured in vitro and fixed for the evaluation of CM, 49 oocytes (57.6%) exhibited complete CM and 36 oocytes(42.4%) exhibited incomplete CM. From 49 oocytes with complete CM, 30 (61.2%) presented a high/positively scoring ZP-BF and 19 (38.8%) presented a low/negatively scoring ZP-BF. From 36 oocytes with incomplete CM, 18 (50%) presented a high/positively scoring ZP-BF and 18 (50%) presented a low/negatively scoring ZP-BF. The correlation between CM and ZP-BF high/positive and low/negative scores was not statistically significant (*P *= 0.42) and showed a low Pearson's correlation coefficient (r = 0.11). Table 3 summarizes the results.

**Table 3:** The correlation between cytoplasmic maturation (CM) in MII oocytes and zona pellucida birefringence intensity (ZP-BF)

CM level Score	ZP-BF Score		Total
	high/positive	low/negative	
Complete CM	30(61.2%)	19(38.8%)	49
Incomplete CM	18(50%)	18(50%)	36
Total	48(56.5%)	37(43.5%)	85

Chi-squared value = 0.65, *P *= 0.42; Pearson's correlation coefficient r = 0.11

The analysis using the numerical values of the scores did not show significant difference in ZP-BF between the group of oocytes that reached CM (2.0 ± 6.8) and the group of oocytes did not reach CM (1.9 ± 6.4; *P *> 0.05). Similarly, in the subgroup of oocytes that presented high/positive ZP-BF, the ZP numerical values of the score was not significantly different in oocytes that reached CM (6.1 ± 3.9) than in the oocytes that did not (6.1 ± 4.4, *P *> 0.05). However, in the subgroup of oocytes that presented low/negative ZP-BF, the ZP numerical values of the score was significantly greater in oocytes that reached CM (-6.2 ± 2.3) than in the oocytes that did not reach CM (-2.8 ± 2.5; *P *= 0.003). Table 4 summarizes the results.

**Table 4:** The correlation between cytoplasmic maturation (CM) in MII oocytes and zona pellucida birefringence intensity (ZP-BF)

CM level Score	ZP-BF Score		
	Total	high/positive	low/negative
Complete CM	2.0 ± 6.8*	6.1 ± 3.9^§^	-6.2 ± 2.3^¶^
Incomplete CM	1.9 ± 5.8*	6.1 ± 4.4^§^	-2.8 ± 2.5^¶^
Total	1.9 ± 6.4	6.1 ± 4.0	-4.5 ± 3.2

The analysis using the numerical values of the ZP scores. Data are reported as means ± SD.

*P > 0.05, ^§^*P *> 0.05, ^¶^*P *= 0.003

## Discussion

In the last decade, ZP-BF intensity has been recognized as a noninvasive test of oocyte quality. However, few studies have analysed its correlation with in vitro oocyte maturation. Incorporation of this new technology into an ICSI program could significantly change the routine manipulation of oocytes, with either positive or negative repercussions.

The complex structure of the ZP forms during the coordinated secretion of glycoproteins during oogenesis [10,11]. However, it has been also demonstrated that the granulosa cells of human ovarian follicles are responsible for the production of ZP proteins during folliculogenesis [12]. At the molecular level, the ZP consists of a 3-dimensional structure composed of 4 protein filaments, ZP1, ZP2, ZP3 and ZP4. A positive correlation between the ZP and oocyte quality could be due, in theory, to the orientation of a perfect connection between the granulosa cells and the oocyte by the ZP. A negative correlation could indicate subfertility because a reduction in the expression of the ZP proteins could provoke an insufficiency in the oocyte maturation process during folliculogenesis by interfering in the biochemical communication between granulosa cells and the ooplasm.

Gook et al. [13] have demonstrated that ZP protein levels increase in the cytoplasmic granulosa cells during follicular development. The presence of these ZP proteins has been also detected in primordial follicles during oogenesis. An effective granulose cell-oocyte signaling may depend on the presence of a functional, highly structured zona. A robust zona with high-order structured fibers probably reflects the healthiness of an oocyte and its full maturation to methaphase II. The birefringence is one the properties of the human ZP that can indicate the formation of the ordered structures of biomolecules in the ZP during oocyte maturation. Polarizing optical system has been used to assess crystalline or paracrystalline structures qualitatively and quantitatively. However, no birefringence change did not mean that other structure changes did not occurred in the ZP. In addiction, previous studies indicate that properties of the zona layers might reflect the history of the oocyte cytoplasmic maturation, whereas different culture conditions may affect the ZP architecture [7,14].

The observation of ZP birefringence (ZP-BF) intensity with a polarized microscope has been proposed to identify oocyte competence and subsequent ART outcomes. Montag et al. [5] have shown that the selection of oocytes based on ZP-BF intensity (high or low) significantly improved implantation and pregnancy rates in a prospective study. Ebner et al. [15] have shown that a high/positive and low/negative score based on the birefringence of the inner zona layer was a strong predictor of blastocyst formation but not of embryo quality or pregnancy. Madaschi et al. [6] have observed an influence of ZP-BF intensity on ICSI clinical outcome after splitting the transferred embryos according to ZP-BF status. For the group in which only embryos derived from high ZP-BF oocytes were transferred, implantation and pregnancy rates were considerably higher and the miscarriage rate lower. Using a different technique (intensity of the zona inner layer retardance), Shen et al. [16] have observed, in a retrospective study, that the retardance magnitude and thickness of the ZP inner layer were higher in oocytes that resulted in pregnancies compared to those that did not. Rama Raju et al. [17] have demonstrated a higher rate of embryonic development to the blastocyst stage when the retardance of the ZP inner layer was >3 nm compared to oocytes with retardance of <3 nm. However, Çiray et al. [18] have not observed a significant difference between the mean of retardance of the ZP inner layer and embryo quality.

The biological reason for ZP-BF intensity as a predictive factor for oocyte quality or good embryonic development is still poorly understood. A high/positively scoring ZP-BF could be the result of an optimal formation of protein structures and the consequent complete oocyte maturation (nuclear and cytoplasmic). In this study, we evaluated whether the integrity of the ZP structure could reflect the ideal cytoplasmic maturation. Through the use of an in vitro-matured oocyte model, we evaluated the correlation between the ZP-BF intensity and the process of oocyte maturation. However, the materials used presented some limitations: 1. Cycles with a reduced number of MII oocytes (<6) were not included because in vitro-matured oocytes could be used for ICSI procedure (and not fixed for CM evaluation); 2. Oocytes from ovarian stimulation cycles when cumulus-free oocytes are matured in media used for conventional IVF may not present the same cytoplasmic characteristics as those obtained from non-stimulated cycles or matured in vivo; 3. GV oocytes represent an already compromised group of oocytes retrieved from follicles that failed to complete maturation in vivo in response to hormonal stimulation. Moreover, the oocytes were denuded of cumulus cells in preparation for ICSI before culture. Cumulus cells are the production site of steroids, growth factors, proteins and other compounds that contribute to the cytoplasmic maturation of oocytes. Beneficial effects of cumulus cells on microtubule dynamics and/or chromatin stability, oocyte maturation and early embryonic development have been reported in many species, including humans [1]. In addition, using CGs as an indicator of CM further limits the value of the study as a) it is shown that the migration of the cortical granules from large clusters in the immature oocyte towards cytoplasmic membrane at maturity can be compromised to some extent by IVM process, and b) the movement of CGs in cytoplasm are affected by culture condition, and cytoplasmic maturity might not fully achieved in the IVM oocytes.

In the present study, a total of 58% of the GV oocytes reached the MII stage with the use of a simple culture medium, i.e., without gonadotropin and steroid supplementation. In a revision on oocyte maturation, Trounson et al. [19] have reported reported a variation of 38% to 84% in the numbers of oocytes from unstimulated cycles reaching full NM after in vitro maturation. Our data showed that the ZP-BF intensity did not alter with the attainment of NM; therefore, the correlation between NM and ZP-BF was low (Table 1). Braga et al. [7] have also observed that during the in vitro spontaneous maturation of oocytes, the ZP-BF remains unaffected.

We did not confirm the hypothesis of a correlation between CM and a high/positive ZP birefringence (Table 2), at least with respect to in vitro-matured GV oocytes from stimulation cycles. Although NM can be accomplished easily in vitro, a concomitant maturation of cytoplasm does not seem to occur properly (i.e., there is a high level of asynchrony between NM and CM). Moreover, in patients with a low number of mature oocytes on the day of retrieval, these GV oocytes could be used to increase the number of embryos obtained. In this situation, the analysis of ZP-BF intensity was not a useful tool for oocyte selection.

## Conclusion

In conclusion, our results demonstrated an absence of a relationship between high/positive and low/negative ZP-BF score and in vitro nuclear and cytoplasmic maturation of oocytes from stimulated cycles. To our knowledge, this study is the first that evaluated the ZP birefringence high/positive and low/negative score and its relationship with oocyte CM.

## Competing interests

The authors declare that they have no competing interests.

## Authors' contributions

CGP was responsible for designing and coordinating the study. All authors were responsible for data collection, data analysis, and data interpretation in the manuscript. CGP, JBAO and JF were responsible for the statistical work and for writing the manuscript. JF was responsible for reviewing the manuscript. All authors read and approved the final manuscript.
